# Remark on the Cauchy problem for the evolution *p*-Laplacian equation

**DOI:** 10.1186/s13660-017-1449-1

**Published:** 2017-08-01

**Authors:** Liangwei Wang, Jngxue Yin, Jinde Cao

**Affiliations:** 1 School of Mathematics and Statistics, Chongqing Three Gorges University, No. 666, Tian Xing Road, Wanzhou District, Chongqing, 404100 China; 2 School of Mathematical Sciences, South China Normal University, No. 55, West of Zhong Shan Road, Tianhe District, Guangzhou, 510631 China; 3 School of Mathematics, and Research Center for Complex Systems and Network Sciences, Southeast University, No. 2, Southeast University Road, Jiangning District, Nanjing, 210996 China

**Keywords:** 35B40, 35K65, chaos, evolution *p*-Laplacian equation, Cauchy problem, propagation estimate, decay estimate

## Abstract

In this paper, we prove that the semigroup $S(t)$ generated by the Cauchy problem of the evolution *p*-Laplacian equation $\frac{\partial u}{\partial t}-\operatorname{div}(|\nabla u|^{p-2}\nabla u)=0$ ($p>2$) is continuous form a weighted $L^{\infty}$ space to the continuous space $C_{0}(\mathbb{R}^{N})$. Then we use this property to reveal the fact that the evolution *p*-Laplacian equation generates a chaotic dynamical system on some compact subsets of $C_{0}(\mathbb{R}^{N})$. For this purpose, we need to establish the propagation estimates and the space-time decay estimates for the solutions first.

## Introduction

In this paper, we consider the Cauchy problems of the evolution *p*-Laplacian equation

1.1 $$\begin{aligned}& \frac{\partial u}{\partial t}-\operatorname{div}\bigl(|\nabla u|^{p-2}\nabla u \bigr)=0 \quad\text{in }(0,\infty)\times\mathbb{R}^{N}, \end{aligned}$$

1.2 $$\begin{aligned}& u(x,0)=u_{0}\quad\text{in }\mathbb{R}^{N}, \end{aligned}$$

where $p>2$ and the nonnegative initial value $u_{0}$ belongs to the weighted $L^{\infty}$ space $W_{\sigma}(\mathbb{R}^{N})\equiv\{\varphi; |x|^{\sigma}\varphi(x)\in L^{\infty}(\mathbb{R}^{N})\}$ with the norm $\| \varphi(\cdot)\|_{W_{\sigma}(\mathbb{R}^{N})}=\||\cdot|^{\sigma}\varphi (\cdot)\|_{L^{\infty}(\mathbb{R}^{N})}$.

The evolution *p*-Laplacian equation, as an important class of parabolic equations, comes from the compressible fluid flows in a homogeneous isotropic rigid porous medium. Comparing to the classical linear heat equation, this equation, to a certain extent, reflects even more exactly physical reality [1, 2]. So the studies of this equation have attracted a large number of mathematicians and remarkable progress has been achieved [3]. Among all of the progress, the semigroup method given by Bénilan and Véron [4, 5] is a successful and effective method to treat the evolution *p*-Laplacian equation.

Using the concepts of dynamical systems to study partial differential equations has also attracted much attention in recent decades. Such concepts, like orbit, *ω*-limit, attractor and chaos, were introduced to investigate the finite dimensional instances of dynamical systems of ordinary differential equations. In 2002, it was Vázquez and Zuazua [6] who first successfully used the *ω*-limit set of the rescaled solutions $u(t^{\frac{1}{2}}\cdot,t)$ to study the complicated asymptotic behavior of solutions for the problem (1.1)-(1.2). Subsequently, we [7–10] investigated the complicated asymptotic behavior of solutions of the porous medium equation by using the *ω*-limit set of the rescaled solutions $t^{\frac{\mu}{2}}u(t^{\beta}\cdot,t)$ with $0<\mu, \beta <\infty$. Using *ω*-limit set to research other partial differential equations, one can refer to [11–14].

The theory of chaos on some partial differential equations has also been well developed since the pioneering work of Li, Wiggins, Shatah and McLaughlin; see [15, 16]. Li [17] revealed the fact that around the Silnikov homoclinic orbits, the existence of chaos on Euler equations can be proved by constructing horseshoe. Cazenave, Dickstein and Weisslerit [18] found that the discrete dynamical system generated by the heat equation in some sense is an example of chaos. In 2005, Battellia and Fečkan proved the existence of homoclinic and chaotic solutions of beam equations in [19]. In 2008, Lan and Li [20] found that the numerical Melnikov integral can be used as a tool for both predicting and controlling chaos on Euler equations. The chaos theory on other partial differential equations, one can see [21–24].

Inspired by the above papers, especially by [18], we focus our attention on the semigroup and the chaos theory for the evolution *p*-Laplacian equation. To overcome the difficulties caused by the degeneracy and nonlinearity of this equation, we first establish the propagation estimate and the decay estimate for the solutions of the problem of (1.1)-(1.2). By using the propagation estimate and the decay estimate of the solutions $u(x,t)$, we see that the semigroup $S(t)$ generated by the evolution *p*-Laplacian equation is continuous from the compact set $B^{\sigma,+}_{M}$ to the space $C_{0}(\mathbb{R}^{N})$, where

$$B^{\sigma,+}_{M} \equiv\bigl\{ \varphi\in W_{\sigma}\bigl( \mathbb{R}^{N}\bigr); \|\varphi\|_{W_{\sigma }(\mathbb{R}^{N})}\leq M \text{ and } \varphi\geq0\bigr\} $$

with the weak-star topology of $W_{\sigma}(\mathbb{R}^{N})$. Then using the definition of chaos, the commutative relation between the delation operator $D^{\sigma }_{\lambda}$ and the semigroup $S(t)$, we find that, for any fixed $\lambda>1$, the map

$$F^{\sigma}_{\lambda}\equiv D^{\sigma}_{\lambda}S\bigl( \lambda^{2}-1\bigr) $$

defined on compact set $S(1)B^{\sigma,+}_{M}$ is chaotic. Here the delation operator $D^{\sigma}_{\lambda}$ is defined as

$$D^{\sigma}_{\lambda}\varphi(x)=\lambda^{\frac{2\sigma}{\sigma(p-2)+p}} \varphi\bigl( \lambda^{\frac{2}{\sigma(p-2)+p}}x\bigr) $$

for $\varphi\in L^{1}_{\mathrm{loc}}(\mathbb{R}^{N})$.

The rest of this paper is organized as follows. In the next section, we give some definitions and some propositions of the solutions for the problem (1.1)-(1.2). Section 3 is devoted to giving the propagation speed estimate and decay estimate for the solutions of problem (1.1)-(1.2). The continuity of the semigroup $S(t)$ is consider in Section 4. We reveal the fact that the problem (1.1)-(1.2) generates a chaotic dynamical system on certain compact subsets of $C_{0}(\mathbb{R}^{N})$ in Section 5.

## Preliminaries

In this section, we give some definitions and present some propositions of solutions for the problem (1.1)-(1.2). We first present the definition of chaos. Although there has been no universally accepted mathematical definition of chaos, the popular text by Devaney [25] isolates three components as being the essential features of chaos. They are formulated for a continuous map $F: X\rightarrow X$ on some metric space $(X,d)$. The first of Devaney’s three conditions is that *F* is transitive; that is, for all non-empty open subsets *U* and *V* of *X* there exists a natural number *k* such that

$$F^{k}(U)\cap V\neq \emptyset. $$

In a certain sense, transitivity is an irreducibility condition. The second of Devaney’s conditions is that the periodic points of *F* form a dense subset of *X*. The final condition is called sensitive dependence on initial conditions; *F* verifies this property if there is a $\delta>0$ such that, for every point $x \in X$ and every neighborhood Ω of *x*, there exist a point $y\in\Omega$ and a nonnegative integer *k* such that

$$d\bigl(F^{k}(x),F^{k}(y)\bigr)>\delta. $$

This sensitivity condition captures the idea that in chaotic systems minute errors in experimental readings eventually lead to large scale divergence. Sensitive dependence on initial conditions is thus widely understood as being the central idea in chaos.

### Definition 2.1

Devaney’s definition of chaos [25]

Let $(X,d)$ be a metric space. A continuous map

$$F: X\to X $$

is said to be chaotic on *X* if

1. *F* is transitive;
2. the periodic points of *F* are dense in *X*;
3. *F* has sensitive dependence on initial conditions.

To discuss chaotic dynamical system in the evolution *p*-Laplacian equation, we need to adopt some concepts as that in [3, 26, 27]. For $f\in L^{1}_{\mathrm{loc}}(\mathbb{R}^{N})$, we define

$$ |\!|\!|f|\!|\!|_{r}=\sup_{R\geq r}R^{-\frac{N(p-2)+p}{p-2}} \int_{\{|x|\leq R\} }\big|f(x)\big|\,\mathrm{d}x $$

and

$$ \ell(f)=\lim_{r\rightarrow\infty}|\!|\!|f|\!|\!|_{r}. $$

The space *X* is given by

$$ X\equiv\bigl\{ \varphi\in L^{1}_{\mathrm{loc}}\bigl( \mathbb{R}^{N}\bigr); |\!|\!|\varphi|\!|\!|_{1}< \infty \bigr\} $$

with the norm $|\!|\!|\cdot|\!|\!|_{1}$. Hence it is a Banach space. The space $X_{0}$ is defined as

$$ X_{0}\equiv\bigl\{ \varphi\in X; \ell(\varphi)=0\bigr\} . $$

The weak solutions of the problem (1.1)-(1.2) is defined as follows.

### Definition 2.2

For $u_{0}\in X$, a measurable function $u=u(x,t)$ defined in $Q_{T}=\mathbb {R}^{N}\times (0,T)$, $T>0$, is a weak solution of the problem (1.1)-(1.2) if

$$u\in C_{\mathrm{loc}}\bigl((0,T); L^{1}_{\mathrm{loc}}\bigl( \mathbb{R}^{N}\bigr)\bigr)\cap L^{p}_{\mathrm{loc}}\bigl(0,T; W^{1,p}_{\mathrm{loc}}\bigl(\mathbb{R}^{N}\bigr)\bigr) $$

and for every test function $\varphi\in C_{0}^{\infty}(Q_{T})$, the following identity holds:

$$\iint_{Q_{T}}\bigl(u\varphi_{t}-|\nabla u|^{p-2}\nabla u\cdot\nabla\varphi\bigr)\, \mathrm{d}x \,\mathrm{d}t=0, $$

and $u(x,t)$ satisfies the initial-value equation (1.2) in the following sense:

$$ u(x,t)\rightarrow u_{0}(x) \quad\text{in } L^{1}_{\mathrm{loc}} \bigl(\mathbb{R}^{N}\bigr) $$

as $t\rightarrow0$.

The existence and uniqueness of weak solution of the problem (1.1)-(1.2) for the initial value $u_{0}\in X$ is shown in [3, 26], and these solutions satisfy the following proposition.

### Proposition 2.1

[3, 26, 28]

*For every* $u_{0}\in X$, *there exist a time* $T=T(u_{0})$ *and a weak solution* $u(x,t)$ *of the problem* (1.1)-(1.2) *in* $Q_{T}$. *Moreover*, *for*

2.1 $$ 0< t\leq T(u_{0})=C\ell(u_{0})^{-(p-2)}, $$

*the solutions* $u(x,t)$ *are Hölder continuous in* $Q_{T}\equiv(0, T)\times\mathbb{R}^{N}$ *and satisfy the following estimates*:

2.2 $$ \big|\!\big|\!\big|u(\cdot,t)\big|\!\big|\!\big|_{r}\leq C|\!|\!|u_{0} |\!|\!|_{r} $$

*and*

2.3 $$ \big|u(x,t)\big|\leq Ct^{-\frac{N}{N(p-2)+p}}R^{\frac{p}{p-2}}|\!|\!|u_{0} |\!|\!|_{r}^{\frac{p}{N(p-2)+p}} \quad\textit{if } r\leq R \textit{ and } |x|\leq R, $$

*where* $B_{R}$ *is the closed ball in* $\mathbb{R}^{N}$ *with the radius* *R*.

From the above proposition, the following proposition can easily been proved; see [26, 29].

### Proposition 2.2

[26, 27]

*Let* $u(x,t)$ *be the nonnegative weak solution of the problem* (1.1)*–*(1.2). *Given* $x_{0}\in\mathbb{R}^{N}$, *if*

$$B(x_{0})=\sup_{R>0}R^{-\frac{N(p-2)+p}{p-2}} \int_{|x_{0}-y|< R} {u_{0}(y)\,\mathrm{d}y}< \infty, $$

*then*, *for all* $0< t\leq C B(x_{0})^{-(p-2)}$,

$$u(x_{0},t)=0. $$

### Proposition 2.3

[27]

*If the initial value* $u_{0}\in X_{0}$, *one can easily see that* $T(u_{0})=\infty$ *and thus these solutions are global*. *Moreover*, *the evolution* *p*-*Laplacian equation generates a bound semigroup in* $X_{0}$ *given by*

2.4 $$ S(t): u_{0}\rightarrow u(x,t). $$

*Moreover*, *if* $1\leq q\leq\infty$ *and* $u_{0}\in L^{q}(\mathbb{R}^{N})\subset X_{0}$, *then* $S(t)$ *is a contraction bounded semigroup in* $L^{q}(\mathbb{R}^{N})$.

For $0<\sigma<N$, $\lambda>0$ and $u_{0}\in X_{0}$, the space-time dilation $\Gamma^{\sigma}_{\lambda}$ is defined as

$$ \Gamma^{\sigma}_{\lambda}\bigl[S(t)u_{0} \bigr](x)=D^{\sigma}_{\lambda}\bigl[S\bigl(\lambda^{2} t \bigr)u_{0}\bigr](x)=\lambda^{\frac{2\sigma}{\sigma(p-2)+p}}u\bigl(\lambda^{\frac {2}{\sigma(p-2)+p}}x, \lambda^{2} t\bigr), $$

where $S(t)$ is the semigroup given by (2.4) and the dilation $D^{\sigma}_{\lambda}$ is given by

$$ D^{\sigma}_{\lambda}\varphi(x)=\lambda^{\frac{2\sigma}{\sigma(p-2)+p}} \varphi\bigl( \lambda^{\frac{2}{\sigma(p-2)+p}}x\bigr) $$

for $\varphi\in L^{1}_{\mathrm{loc}}(\mathbb{R}^{N})$. In this paper, we consider our problem in the $L^{\infty}$ weight space $W_{\sigma}(\mathbb{R}^{N})\equiv\{\varphi\in L^{1}_{\mathrm{loc}}(\mathbb {R}^{N}); |x|^{\sigma}\varphi(x)\in L^{\infty}(\mathbb{R}^{N})\} $ with $0<\sigma<N$. We equip this space with the norm $\|\varphi\|_{W_{\sigma}(\mathbb {R}^{N})}=\||\cdot|^{\sigma}\varphi(\cdot)\|_{L^{\infty}(\mathbb{R}^{N})}$. Hence it is a Banach space. Meanwhile, one easily verifies that, for $0<\sigma<N$, $W_{\sigma}(\mathbb{R}^{N})\subset X_{0}$. The closed convex set

$$B_{M}^{\sigma,+}\equiv\bigl\{ \varphi\in W_{\sigma}\bigl( \mathbb{R}^{N}\bigr); \|\varphi\| _{W_{\sigma}(\mathbb{R}^{N})}\leq M \text{ and } \varphi\geq0\bigr\} $$

with the weak-star topology of $W_{\sigma}(\mathbb{R}^{N})$ is compact and separable. Thus it can be meterizable. We use the symbol $d^{\sigma ,*}_{M}$ to denote this metric. So, for all $M\geq0$, the metric space $(B_{M}^{\sigma,+}, d^{\sigma,*}_{M})$ is compact, hence complete and separable.

In the rest of this section, we study the relation between the semigroup operator $S(t)$ and the dilation operator $D^{\sigma}_{\lambda}$. Suppose $u(x,t)$ is a weak solution of the problem (1.1)-(1.2) with initial value $u_{0}\in X_{0}$. Let

2.5 $$ v(x,t)=\Gamma_{\lambda}^{\sigma}\bigl[S(t)u_{0} \bigr](x)=\lambda^{\frac{2\sigma }{\sigma(p-2)+p}}\bigl(S\bigl(\lambda^{2}t\bigr) u_{0}\bigr) \bigl(\lambda^{\frac{2}{\sigma(p-2)+p}}x\bigr). $$

So

$$\frac{\partial v}{\partial t}=\hbox{div}\bigl(|\nabla v|^{p-2}\cdot\nabla v\bigr). $$

This mean that $v(x,t)$ is a weak solution of the following Cauchy problem:

$$ \textstyle\begin{cases} \frac{\partial v}{\partial t}=\mathrm{div}(|\nabla v|^{p-2}\cdot\nabla v)&\text{in } \mathbb{R}^{N}\times(0,\infty),\\ v(x,0)=\lambda^{\frac{2\sigma}{\sigma(p-2)+p}}u_{0}(\lambda^{\frac{2}{\sigma (p-2)+p}}x) =D^{\sigma}_{\lambda}u_{0}(x) &\text{in }\mathbb{R}^{N}. \end{cases} $$

Note that

$$\big\| D^{\sigma}_{\lambda}u_{0}\big\| _{W^{\sigma}(\mathbb{R}^{N})}= \|u_{0}\|_{W^{\sigma }(\mathbb{R}^{N})}. $$

So, $u_{0}\in W^{\sigma}(\mathbb{R}^{N})$, hence

$$v(x,t)=S(t) \bigl(D^{\sigma}_{\lambda}u_{0}\bigr) (x). $$

Then we get the following commutative relation between the semigroup operator $S(t)$ and the dilation operator $D^{\sigma}_{\lambda}$:

2.6 $$ \Gamma^{\sigma}_{\lambda}\bigl[S(t)u_{0} \bigr]=D^{\sigma}_{\lambda}\bigl[S\bigl(\lambda^{2} t \bigr)u_{0}\bigr]=S(t)\bigl[D^{\sigma}_{\lambda}u_{0} \bigr]. $$

For any fixed $\lambda>1$ and $0<\sigma<N$, we now define the map $F^{\sigma}_{\lambda}: S(1)B^{\sigma,+}_{M}\to S(1)B^{\sigma,+}_{M}$ as

$$F^{\sigma}_{\lambda}\equiv D^{\sigma}_{\lambda}S\bigl( \lambda^{2}-1\bigr)=S\biggl(1-\frac {1}{\lambda^{2}}\biggr)D^{\sigma}_{\lambda}. $$

## Some estimates

In this section, we first estimate the propagation speed of solutions for Problem (1.1)-(1.2) with the nonnegative initial value $u_{0}\in W_{\sigma}(\mathbb{R}^{N})$. For this purpose, we need some concepts. Let

$$d(x)\equiv\sup\bigl\{ R; u_{0}(y)=0 \text{ a.e. in } B_{R}(x) \bigr\} $$

be the distance from *x* to the support of $u_{0}$ and let us introduce the following symbol to denote the positive set of $u(x,t)$ at time *t*:

$$\Omega(t)\equiv\bigl\{ x\in\mathbb{R}^{N}; u(x,t)>0\bigr\} . $$

We also define the *ρ*-neighborhood of the set $\Omega(t)$ as

$$\Omega_{\rho}(t)\equiv\bigl\{ x\in\mathbb{R}^{N}; d\bigl(x, \Omega(t)\bigr)< \rho\bigr\} , $$

where $d(x,\Omega(t))$ is the distance from *x* to $\Omega(t)$.

### Theorem 3.1

Propagation estimate

*Suppose* $0<\sigma<N$ *and the initial value* $u_{0}\in W_{\sigma}^{+}(\mathbb{R}^{N})$, *i*.*e*., $u_{0}\geq0$ *and* $u_{0}\in W_{\sigma}(\mathbb{R}^{N})$. *Let* $u(x,t)$ *be a nonnegative weak solution of the problem* (1.1)-(1.2). *For any* $0\leq t_{1}< t_{2}<\infty$, *then*

$$\Omega(t_{2})\subset\Omega_{\rho(t_{2}-t_{1})}(t_{1}), $$

*where* $\rho(t_{2}-t_{1})=C(t_{2}-t_{1})^{\frac{1}{\sigma(p-2)+p}}\|u_{0}\|_{W_{\sigma}(\mathbb{R}^{N})}^{\frac{p-2}{\sigma(p-2)+p}}$.

### Proof

Without loss of generality, we restrict our consideration to the case $t_{1}=0$. Assume $x_{0}\in\mathbb{R}^{N}$ with $d(x_{0})>0$. Note first that, if $R< d(x_{0})$, then

3.1 $$ \int_{B_{R}(x_{0})}u_{0}(y)\,\mathrm{d}y=0. $$

For any $r\geq0$, let $R=d(x_{0})+r\geq d(x_{0})$. If $|x_{0}|<2R$, then

$$B_{R}(x_{0})\subset B_{3R}(0). $$

Therefore,

3.2 $$ \begin{aligned}[b] R^{-\frac{N(p-2)+p}{p-2}} \int_{B_{R}(x_{0})}u_{0}(y)\,\mathrm{d}y &\leq C \|u_{0}\|_{W_{\sigma}(\mathbb{R}^{N})}R^{-\frac{N(p-2)+p}{p-2}} \int _{B_{3R}(0)}|y|^{-\sigma}\,\mathrm{d}y \\ &=C\|u_{0}\|_{W_{\sigma}(\mathbb{R}^{N})}R^{-\frac{p}{p-2}-\sigma}\leq C\| u_{0} \|_{W_{\sigma}(\mathbb{R}^{N})}d(x_{0})^{-\frac{p}{p-2}-\sigma}. \end{aligned} $$

If $|x_{0}|\geq2R$, then, for any $y\in B_{R}(x_{0})$,

$$|y|\geq|x_{0}|-R\geq R. $$

So for $0<\sigma<N$, we have

$$|y|^{-\sigma}\leq R^{-\sigma}. $$

Therefore,

$$ \begin{aligned} R^{-\frac{N(p-2)+p}{p-2}} \int_{B_{R}(x_{0})}u_{0}(y)\,\mathrm{d}y &\leq C \|u_{0}\|_{W_{\sigma}(\mathbb{R}^{N})} R^{-\frac{N(p-2)+p}{p-2}-\sigma} \int_{B_{R}(x_{0})}\mathrm{d}y \\ &=C\|u_{0}\|_{W_{\sigma}(\mathbb{R}^{N})}R^{-\frac{p}{p-2}-\sigma}\leq C\| u_{0} \|_{W_{\sigma}(\mathbb{R}^{N})}d(x_{0})^{-\frac{p}{p-2}-\sigma}. \end{aligned} $$

Combining this with (3.1) and (3.2), we have

$$ B(x_{0})=\sup_{R>0} R^{-\frac{N(p-2)+p}{p-2}} \int_{B_{R}(x_{0})}u_{0}(y)\,\mathrm{d}y \leq C \|u_{0}\|_{W_{\sigma}(\mathbb{R}^{N})}d(x_{0})^{-\frac{(p-2)\sigma+p}{p-2}}. $$

Therefore, Proposition 2.2 implies

$$u(x_{0},t)=0 \quad\text{for all } 0\leq t\leq C\|u_{0} \|_{W_{\sigma}(\mathbb{R}^{N})}^{-(p-2)}d(x_{0})^{(p-2)\sigma+p}. $$

This means

$$\Omega(t)\subset\Omega_{\rho(t)}(0), $$

where $\rho(t)=C\|u_{0}\|_{W_{\sigma}(\mathbb{R}^{N})}^{\frac{p-2}{\sigma(p-2) +p}}t^{\frac{1}{(p-2)\sigma+p}}$. So we complete the proof of this theorem. □

In the rest of this section, we pay our attention on the properties of the semigroup $S(t)$.

### Theorem 3.2

*Let* $\omega\in C(\mathbb{R}^{N}\setminus\{0\})$ *be a homogeneous function of degree* 0. *Suppose* $0<{\sigma<N}$ *and set* $u_{0}(x)=|x|^{-\sigma}\omega(x)$. *It follows that*

3.3 $$ S(t)u_{0}(x)=t^{-\frac{\sigma}{\sigma(p-2)+p}}g\bigl(t^{-\frac{1}{\sigma(p-2)+p}}x \bigr), $$

*where* $g(x)\in C^{\alpha}(\mathbb{R}^{N})$ *and* $|x|^{\sigma}g(x)-\omega (x)\rightarrow0$ *as* $|x|\rightarrow\infty$.

### Proof

From (2.6) and the definition of the initial value $u_{0}$, we have

3.4 $$ \begin{aligned}[b] \Gamma^{\sigma}_{\lambda} \bigl[S(s)u_{0}(x)\bigr]&=\lambda^{\frac{2\sigma}{\sigma (p-2)+p}}\bigl[S\bigl( \lambda^{2} s\bigr)u_{0}\bigr]\bigl(\lambda^{\frac{2}{\sigma(p-2)+p}}x \bigr) \\ &=S(s)\bigl[\lambda^{\frac{2\sigma}{\sigma(p-2)+p}}u_{0}\bigl(\lambda^{\frac {2}{\sigma(p-2)+p}} \cdot\bigr)\bigr](x) =S(s)u_{0}(x). \end{aligned} $$

First notice that, for $0<\sigma<N$,

$$ \|u_{0}\|_{r}=\sup_{R\geq r}R^{-\frac{N(p-2)+p}{p-2}} \int_{B_{R}}\big|u_{0}(x)\big|\, \mathrm{d}x\leq Cr^{-\sigma-\frac{p}{p-2}}\rightarrow0\quad\text{as } r\rightarrow\infty. $$

So,

$$u_{0}\in X_{0}. $$

Therefore,

$$ S(s)u_{0}\in C^{\frac{\alpha}{2}, \alpha}\bigl((0,\infty)\times \mathbb{R}^{N}\bigr) $$

for some $0<\alpha<1$; see [26, 27]. In particular,

$$ S(1)u_{0}(x)\in C^{\alpha}\bigl(\mathbb{R}^{N}\bigr). $$

Now taking $s=1$, $\lambda=t^{\frac{1}{2}}$ and $g(x)=S(1)u_{0}(x)$ in the expression (3.4), we obtain

$$ S(t)u_{0}(x)=t^{-\frac{\sigma}{\sigma(p-2)+p}}g\bigl(t^{-\frac{1}{\sigma(p-2)+p}}x\bigr) $$

and

$$g(x)\in C^{\alpha}\bigl(\mathbb{R}^{N}\bigr). $$

The fact $S(t)u_{0}(x)\in C([0,\infty)\times\mathbb{R}^{N}\setminus\{0,0\} )$ [26, 29] clearly implies that, for $|x|=1$,

$$ t^{-\frac{\sigma}{\sigma(p-2)+p}}g\bigl(t^{-\frac{1}{\sigma (p-2)+p}}x\bigr)=S(t)u_{0}(x) \rightarrow\varphi(x)=|x|^{-\sigma}\omega (x)=\omega(x) $$

as $t\to0$. Let

$$y=t^{-\frac{1}{\sigma(p-2)+p}}x. $$

So

$$|y|\rightarrow\infty\quad\text{as } t\rightarrow0. $$

Therefore,

$$|y|^{\sigma}g(y)-\omega(y)\rightarrow0, $$

as $|y|\rightarrow\infty$. We complete the proof of this theorem. □

### Theorem 3.3

Space-time decay estimates

*Given* $0<\sigma<N$ *and a constant* $M>0$, *there exists a constant* *C* *such that if* $u_{0}\in B^{\sigma,+}_{M}(\mathbb{R}^{N})$, *then*

3.5 $$ S(t)u_{0}(x)\leq C\bigl(t^{\frac{2}{N(p-2)+p}}+|x|^{2} \bigr)^{-\frac{\sigma}{2}}, $$

*for all* $t>0$ *and all* $x\in\mathbb{R}^{N}$.

### Proof

Let

$$\varphi(x) = M|x|^{-\sigma} $$

and

$$g(x)=S(1)\varphi(x). $$

It follows from Theorem 3.2 that there exists a constant *C* such that

$$ \big|g(x)\big|\leq C\bigl(1+|x|^{2}\bigr)^{-\frac{\sigma}{2}}. $$

So by (3.3),

$$ S(t)\varphi(x)\leq C\bigl(t^{\frac{2}{\sigma(p-2)+p}} + |x|^{2} \bigr)^{-\frac {\sigma}{2}}. $$

By the comparison principle [3, 27], we get

$$ S(t)u_{0}(x)\leq S(t)\varphi(x)\leq C\bigl(t^{\frac{2}{\sigma(p-2)+p}} + |x|^{2}\bigr)^{-\frac{\sigma}{2}}. $$

So the proof of this theorem is complete. □

## Continuity of the semigroup

In this section, we first present the fact that the semigroup operator $S(t)$ are continuous from the metric space $B^{\sigma,+}_{M}$ to the space $C_{0}(\mathbb{R}^{N})$, which is the basis of the proof of our main result.

### Theorem 4.1

*For any fixed* $\tau>0$, *let* $0 <\sigma< N$ *and* $M >0$, *then*, *for any* $t >\tau$, $S(t): (B^{\sigma,+}_{M}, d_{M}^{\sigma,*})\rightarrow C_{0}(\mathbb {R}^{N})$ *is continuous*. *In particular*, $S(1)$ *is a continuous map from the metric space* $(B^{\sigma,+}_{M}, d_{M}^{\sigma,*})$ *to the space* $C_{0}(\mathbb{R}^{N})$.

### Proof

Let $\{u_{n}\}_{n\geq1}\subset B^{\sigma,+}_{M}$ and $u_{0}\in B^{\sigma,+}_{M}$ such that

$$d^{\sigma,*}_{M}(u_{n},u_{0})\rightarrow0\quad \text{as } n\rightarrow\infty. $$

Therefore,

4.1 $$ u_{n}\rightarrow u_{0}\quad\text{in } \mathscr{D}'\bigl(\mathbb{R}^{N}\bigr) \text{ as } n \rightarrow\infty. $$

Notice also that

$$\|u_{n}\|_{W_{\sigma}(\mathbb{R}^{N})}\leq M \quad\text{for all } n\geq0. $$

For any $t, R>0$, let

$$R_{1}(t)=R+1+CM^{\frac{p-2}{\sigma(p-2)+p}} t^{\frac{1}{\sigma(p-2)+p}}. $$

So, for all $n\geq0$,

$$ \operatorname{supp}\bigl[(1-\chi_{R_{1}(t)})u_{n}\bigr]\subset \bigl\{ x\in\mathbb{R}^{N}; |x|> R+1+CM^{\frac{p-2}{\sigma(p-2)+p}}t^{\frac{1}{\sigma(p-2)+p}} \bigr\} , $$

where $\chi_{R_{1}(t)}$ is the cut-off function defined on the ball $B_{R_{1}(t)+1}\equiv\{x\in\mathbb{R}^{N}; |x|\leq R_{1}(t)+1\}$ relative to the ball $B_{R_{1}(t)}\equiv\{x\in\mathbb{R}^{N}; |x|\leq R_{1}(t)\}$, *i.e.*,

$$\chi_{R_{1}(t)}(x)\in C_{0}^{\infty}\bigl( \mathbb{R}^{N}\bigr), \quad0\leq\chi _{R_{1}(t)}(x)\leq1 $$

and

$$ \chi_{R_{1}(t)}(x)= \textstyle\begin{cases} 1&\text{for } x\in B_{R_{1}(t)},\\ 0&\text{for } x\notin B_{R_{1}(t)+1}. \end{cases} $$

By Lemma 3.1, we get

$$ \operatorname{supp}\bigl[S(t) (1-\chi_{R_{1}(t)})u_{n}\bigr] \subset\bigl\{ x\in\mathbb{R}^{N}; |x|\geq R+1\bigr\} . $$

This means that the value of $S(t)u_{n}(x)$ in $B_{R}$ is only dependent on the initial value $u_{n}$ in $B_{R_{1}(t)}$. In other words, for $x\in B_{R}$,

4.2 $$ S(t)u_{n}(x)=S(t)[\chi_{{R_{1}(t)}}u_{n}](x). $$

For any $\epsilon>0$, taking the above *R* large enough, the inequality (3.5) clearly implies that, if $|x|\geq R$, then

4.3 $$ \big|S(t)u_{n}(x)\big|< \frac{\epsilon}{3}\quad\text{for all } n \geq0. $$

By (4.1) and the hypothesis $u_{n}, u_{0}\in W_{\sigma}(\mathbb {R}^{N})$, we get, for $1< q<\frac{N}{\sigma}$,

$$\chi_{{R_{1}(t)}}u_{n}\rightharpoonup\chi_{{R_{1}(t)}}u_{0} \quad\text{in } L^{q}\bigl(\mathbb{R}^{N}\bigr). $$

So,

$$S(\tau)[\chi_{{R_{1}(t)}}u_{n}]\rightharpoonup S(\tau)[ \chi_{{R_{1}(t)}}u_{0}]\quad \text{in } L^{q}\bigl( \mathbb{R}^{N}\bigr). $$

In particular,

$$S(\tau)[\chi_{{R_{1}(t)}}u_{n}]\rightarrow S(\tau)[ \chi_{{R_{1}(t)}}u_{0}]\quad \text{in } \mathscr{D}'( \mathbb{R}). $$

From (3.5), we know that there exists a constant $C(\tau)$ such that

$$\big\| S(\tau)[\chi_{{R_{1}(t)}}u_{n}]\big\| _{L^{\infty}(\mathbb{R}^{N})}\leq C(\tau)\quad \text{for all } n\geq0. $$

Therefore,

$$S(\tau)[\chi_{{R_{1}(t)}}u_{n}]\rightarrow S(\tau)[ \chi_{{R_{1}(t)}}u_{0}]\quad \text{weakly-star in } L^{\infty} \bigl(\mathbb{R}^{N}\bigr). $$

From the regularity of the semigroup operator $S(t)$, we obtain, for any fixed $t>\tau>0$,

$$ \big\| S(t)[\chi_{{R_{1}(t)}}u_{n}]-S(t)[\chi_{{R_{1}(t)}}u_{0}] \big\| _{L^{\infty }_{\mathrm{loc}}(\mathbb{R}^{N})}\rightarrow0 $$

as $n\rightarrow\infty$. This implies, via (4.2), that

$$ \big\| S(t)[u_{n}]-S(t)[u_{0}]\big\| _{L^{\infty}(B_{R})}=\big\| S(t)[ \chi_{{R_{1}(t)}}u_{n}]-S(t) [\chi_{{R_{1}(t)}}u_{0}] \big\| _{L^{\infty}(B_{R})}\rightarrow0. $$

By (4.3), there exists an integer *N* such that if $n>N$, then

$$ \begin{gathered} \big\| S(t)[u_{n}]-S(t)[u_{0}]\big\| _{L^{\infty}(\mathbb{R}^{N})} \\ \quad\leq \big\| S(t)[u_{n}]-S(t)[u_{0}]\big\| _{L^{\infty}(B_{R})}+ \big\| S(t)[u_{n}]\big\| _{L^{\infty }(\mathbb{R}^{N}\setminus B_{R})}+\big\| S(t)[u_{0}] \big\| _{L^{\infty}(\mathbb {R}^{N}\setminus B_{R})} \\ \quad< \epsilon. \end{gathered}$$

So we complete the proof of this theorem. □

## Chaotic dynamical system

For any $\lambda> 1$ and $M > 0$, we recall that the map $F^{\sigma }_{\lambda}: S(1)B^{\sigma,+}_{M}\mapsto S(1)B^{\sigma,+}_{M}$ is defined as

$$ F^{\sigma}_{\lambda}=D^{\sigma}_{\lambda}S\bigl( \lambda^{2}-1\bigr). $$

The ideas of the following theorem come from [18]. We also need a lemma which appeared in [30].

### Lemma 5.1

*If* $f: X\rightarrow X$ *is transitive and has dense periodic points*, *then F has sensitive dependence on initial conditions*.

### Theorem 5.1

*If* $\lambda> 1$ *and* $M > 0$, *then the map* $F^{\sigma}_{\lambda}: S(1)B^{\sigma,+}_{M}\mapsto S(1)B^{\sigma,+}_{M}$ *is chaotic*.

### Proof

We first verify that the map $F^{\sigma}_{\lambda}$ is well defined. By Theorem 4.1, the uniqueness theorem for the solution of (1.1)-(1.2) with $u_{0}\in X_{0}$, we see that $S(1)$ is a continuous, injective, surjective map from the compact Hausdorff space $B^{\sigma,+}_{M}$ onto the Hausdorff space $S(1)B^{\sigma,+}_{M}$. So we see that $S(1)$ is a homeomorphism from the compact set $B^{\sigma,+}_{M}$ to $S(1)B^{\sigma,+}_{M}$ by the fact that a continuous, injective, surjective map of the compact Hausdorff space onto the Hausdorff space is a homeomorphism. This means that $S(1)B^{\sigma,+}_{M}$ is a compact set. For any $\varphi\in B^{\sigma ,+}_{M}$, we have

$$F^{\sigma}_{\lambda}\bigl[S(1)\varphi\bigr]=S(1)\bigl[D^{\sigma}_{\lambda} \varphi\bigr]\in S(1)B_{M}^{\sigma,+}. $$

So, $F^{\sigma}_{\lambda}$ is well defined. We will divide the rest proof into four steps.

*1. The map* $F^{\sigma}_{\lambda}$ *is a continuous map on the compact set* $S(1)B_{M}^{\sigma,+}$ *.*

For any sequence $\{v_{k}\}_{k\geq1}\subset B_{M}^{\sigma,+}$ and $v_{0}\in B_{M}^{\sigma,+}$, if

$$S(1)v_{k}\to S(1)v_{0} \quad\text{in } C_{0} \bigl(\mathbb{R}^{N}\bigr) \text{ as } k\rightarrow\infty, $$

then

$$S\bigl(\lambda^{2}\bigr)v_{k}=S\bigl(\lambda^{2}-1 \bigr)\bigl[S(1)v_{k}\bigr]\to S\bigl(\lambda ^{2}-1\bigr) \bigl[S(1)v_{0}\bigr]=S\bigl(\lambda^{2}\bigr)v_{0}\quad \text{in } C_{0}\bigl(\mathbb{R}^{N}\bigr) $$

as $k\rightarrow\infty$. Here we have used the facts that $\lambda>1$ and $S(t)$ is a bounded contractive continuous semigroup in $L^{\infty}(\mathbb{R}^{N})$ for $t>0$ (Proposition 2.3). Therefore,

$$ \begin{gathered} \big\| F^{\sigma}_{\lambda}\bigl[S(1)v_{k}\bigr]-F^{\sigma}_{\lambda}\bigl[S(1)v_{0}\bigr]\big\| _{L^{\infty }(\mathbb{R}^{N})} \\ \quad=\big\| D^{\sigma}_{\lambda}\bigl[S\bigl(\lambda^{2} \bigr)v_{k}\bigr]-D^{\sigma}_{\lambda}\bigl[S\bigl(\lambda ^{2}\bigr)v_{0}\bigr]\big\| _{L^{\infty}(\mathbb{R}^{N})} \\ \quad=\big\| S\bigl(\lambda^{2}\bigr)v_{k}-S\bigl( \lambda^{2}\bigr)v_{0}\big\| _{L^{\infty}(\mathbb{R}^{N})}\to0 \end{gathered}$$

as $k\rightarrow\infty$. This means that the map $F^{\sigma}_{\lambda}$ is a continuous map on the compact set $S(1)B_{M}^{\sigma,+}$.

*2. The periodic points of* $F_{\lambda}^{\sigma}$ *are dense in* $S(1)B^{\sigma,+}_{M}$ *.*

For any $k\in\mathbb{Z}^{+}$, $v\in B^{\sigma,+}_{M}$ and $\lambda>1$, let $v_{k}$ be defined as

$$ v_{k}(x)=\sum_{n=-\infty}^{+\infty} \chi_{n}(x)\lambda^{\frac{2n\sigma k}{\sigma(p-2)+p}}v\bigl(\lambda^{\frac{2nk}{\sigma(p-2)+p}}x\bigr), $$

where

$$ \chi_{n}(x)= \textstyle\begin{cases} 1&\text{if } x\in A_{n}=\{\lambda^{\frac{(2n-1)k}{\sigma (p-2)+p}}\leq|y| < \lambda^{\frac{(2n+1)k}{\sigma(p-2)+p}}\},\\ 0&\text{if } x\notin A_{n}. \end{cases} $$

Note that, for all $k>0$,

$$\|v_{k}\|_{W_{\sigma}(\mathbb{R}^{N})}\leq\|v\|_{W_{\sigma}(\mathbb{R}^{N})}\leq M $$

and

$$\bigl(D^{\sigma}_{\lambda}\bigr)^{k} v_{k}=v_{k}. $$

So

$$v_{k}\in B^{\sigma,+}_{M} $$

and

$$\bigl(F_{\lambda}^{\sigma}\bigr)^{k}\bigl(S(1)v_{k} \bigr) = \bigl(F_{\lambda}^{\sigma }\bigr)^{k-1}\bigl[S(1) \bigl(D^{\sigma}_{\lambda}v_{k}\bigr)\bigr]=\cdots= S(1)\bigl[ \bigl(D^{\sigma}_{\lambda}\bigr)^{k}v_{k}) \bigr]=S(1)v_{k}. $$

Therefore, $S(1)v_{k}$ is a periodic point of $F_{\lambda}^{\sigma}$. Note also that

$$v_{k}(x)=v(x)\quad \text{if }x\in A_{0}. $$

This means that

$$v_{k}\rightarrow v\quad\text{in } \mathscr{D}'\bigl( \mathbb{R}^{N}\setminus\{0\} \bigr) \text{ as } k\rightarrow\infty, $$

hence

$$v_{k}\rightarrow v \quad\text{in } \bigl(B_{M}^{\sigma,+}, d^{\sigma,*}_{M}\bigr) \text{ as } k\rightarrow\infty. $$

Using Theorem 4.1, we get

$$S(1)v_{k}\to S(1)v\quad \text{in } C_{0}\bigl( \mathbb{R}^{N}\bigr) \text{ as } k\rightarrow\infty. $$

This proves that the periodic points of $F_{\lambda}^{\sigma}$ are dense in $S(1)B^{\sigma,+}_{M}$.

*3. The map* $F_{\lambda}^{\sigma}$ *is topologically transitive.*

For any open subsets *U* and *V* of $S(1)B^{\sigma,+}_{M}$, there exist a constant $\varepsilon>0$ and two functions *φ*, $\phi\in B^{\sigma ,+}_{M}$ such that

5.1 $$ B_{\varepsilon}\bigl(S(1)\varphi\bigr)\subset U $$

and

5.2 $$ B_{\varepsilon}\bigl(S(1)\phi\bigr)\subset V. $$

Now let

5.3 $$ U_{0}(x)=\sum_{n=1}^{\infty} \bigl(D^{\sigma}_{\lambda_{2n}^{-1}}\bigl[\chi _{2n}(x)\phi(x)\bigr]+ D^{\sigma}_{\lambda_{2n-1}^{-1}}\bigl[\chi_{2n-1}(x)\varphi(x)\bigr] \bigr), $$

where

$$ \lambda_{n}=\lambda^{2^{n+1}} $$

and $\chi_{n}(x)$ is the cut-off function defined on the set $A_{n}\equiv \{x\in\mathbb{R}^{N}; \lambda^{\frac{-n}{4\sigma(p-2)+4p}}< |x|<\lambda^{\frac{n}{\sigma(p-2)+p}}\}$ relative to the set $A_{n-1}\equiv\{x\in\mathbb{R}^{N}; \lambda^{\frac{-n+1}{\sigma(p-2)+p}}< |x|<\lambda^{\frac{n-1}{\sigma(p-2)+p}}\}$. Notice that, for all $n\geq1$,

5.4 $$ \operatorname{supp}\bigl[D^{\sigma}_{\lambda_{2n}^{-1}}\bigl( \chi_{2n}(x)\phi (x)\bigr)\bigr]\subset\bigl\{ x\in\mathbb{R}^{N}; \lambda^{\frac{2^{2n+1}-2n}{\sigma (p-2)+p}}< |x|< \lambda^{\frac{2^{2n+1}+2n}{\sigma(p-2)+p}}\bigr\} $$

and

5.5 $$ \operatorname{supp}\bigl[D^{\sigma}_{\lambda_{2n-1}^{-1}}\bigl( \chi _{2n-1}(x)\varphi(x)\bigr)\bigr]\subset\bigl\{ x\in \mathbb{R}^{N}; \lambda^{\frac {2^{2n}-2n+1}{\sigma(p-2)+p}}< |x|< \lambda^{\frac{2^{2n}+2n-1}{\sigma(p-2)+p}}\bigr\} . $$

So, for $n\geq1$, from (5.4) and (5.5), we have

$$\operatorname{supp}D^{\sigma}_{\lambda_{2n}^{-1}}\bigl[\chi_{2n}(x) \phi (x)\bigr]\cap\operatorname{supp} D^{\sigma}_{\lambda_{2n+1}^{-1}}\bigl[ \chi_{2n+1}(x)\varphi(x)\bigr]=\emptyset $$

and

$$\operatorname{supp}D^{\sigma}_{\lambda_{2n}^{-1}}\bigl[\chi_{2n}(x) \phi (x)\bigr]\cap\operatorname{supp} D^{\sigma}_{\lambda_{2n-1}^{-1}}\bigl[ \chi_{2n-1}(x)\varphi(x)\bigr]=\emptyset. $$

Here we have used the fact that, if $i>j\geq1$, then

$$2^{i+1}-i>2^{j+1}+j. $$

So

$$U_{0}\in B^{\sigma,+}_{M}. $$

Note also that

$$D^{\sigma}_{\lambda_{2n}}U_{0}=\phi\quad\text{in } A_{2n-1} $$

and

$$D^{\sigma}_{\lambda_{2n+1}}U_{0}=\varphi\quad\text{in } A_{2n} $$

for $n\geq1$. So

$$D^{\sigma}_{\lambda_{2n}}U_{0}\xrightarrow{n\rightarrow\infty} \phi\quad \text{weakly-star in } W_{\sigma}\bigl(\mathbb{R}^{N} \bigr) $$

and

$$D^{\sigma}_{\lambda_{2n+1}}U_{0}\xrightarrow{n\rightarrow\infty} \varphi \quad\text{weakly-star in } W_{\sigma}\bigl(\mathbb{R}^{N} \bigr). $$

It follows from Theorem 4.1 that

$$S(1)D^{\sigma}_{\lambda_{2n}}U_{0}\xrightarrow{n\rightarrow \infty }S(1)\phi\quad\text{in } C_{0}\bigl(\mathbb{R}^{N}\bigr) $$

and

$$S(1)D^{\sigma}_{\lambda_{2n+1}}U_{0}\xrightarrow{n\rightarrow \infty }S(1)\varphi\quad\text{in } C_{0}\bigl(\mathbb{R}^{N} \bigr). $$

Then we conclude from the definition of $F^{\sigma}_{\lambda}$ that

5.6 $$ \bigl(F^{\sigma}_{\lambda} \bigr)^{2^{2n-1}}\bigl[S(1)U_{0}\bigr] =S(1)\bigl[ \bigl(D^{\sigma}_{\lambda}\bigr)^{2^{2n}}U_{0}\bigr] \xrightarrow{n\rightarrow\infty} S(1)\phi \quad\text{in } C_{0}\bigl( \mathbb{R}^{N}\bigr) $$

and

5.7 $$ \bigl(F^{\sigma}_{\lambda} \bigr)^{2^{2n}}\bigl[S(1)U_{0}\bigr] =S(1)\bigl[ \bigl(D^{\sigma}_{\lambda}\bigr)^{2^{2n+1}}U_{0}\bigr] \xrightarrow{n\rightarrow\infty} S(1)\varphi\quad \text{in } C_{0}\bigl( \mathbb{R}^{N}\bigr). $$

So for the above $\varepsilon>0$, there exists $N\in\mathbb{N}$ such that if $n\geq N$, then

$$\bigl(F^{\sigma}_{\lambda}\bigr)^{2^{2n-1}}\bigl[S(1)U_{0} \bigr]\in B_{\varepsilon}\bigl(S(1)\phi \bigr)\subset V $$

and

$$\bigl(F^{\sigma}_{\lambda}\bigr)^{2^{2n}}\bigl[S(1)U_{0} \bigr]\in B_{\varepsilon}\bigl(S(1)\varphi \bigr)\subset U. $$

These results mean that

$$F_{\lambda}^{\sigma}U\cap V\neq\emptyset. $$

So we complete the proof of that $F^{\sigma}_{\lambda}$ is topologically transitive.

So the map $F^{\sigma}_{\lambda}$ is chaotic by Devaney’s definition of chaos and Lemma 5.1, and the proof of this theorem is complete. □

## Conclusion

In this paper, our concern here is the properties of the solutions to the Cauchy problem of the evolution *p*-Laplacian equation with the initial value $u_{0}$ belonging to a weighted $L^{\infty}$ spaces, and we get the following results:

I. The propagation estimate and the decay estimate for the solutions of the problem of (1.1)-(1.2) have been established.
II. The semigroup $S(t)$ generated by the evolution *p*-Laplacian equation is continuous from the compact set $B^{\sigma ,+}_{M}$ to the space $C_{0}(\mathbb{R}^{N})$.
III. The map $F^{\sigma}_{\lambda}$ generated by the evolution *p*-Laplacian equation is chaotic on the compact subset $S(1)B^{\sigma ,+}_{M}$ of $C_{0}(\mathbb{R}^{N})$.

In future work, we hope to continue to study the properties of solutions for the Cauchy problem of the evolution *p*-Laplacian equation with the initial value $u_{0}$ belonging to other Banach spaces, especially the unbounded spaces.
